# Jiedu Sangen Decoction Inhibits the Invasion and Metastasis of Colorectal Cancer Cells by Regulating EMT through the Hippo Signaling Pathway

**DOI:** 10.1155/2019/1431726

**Published:** 2019-06-25

**Authors:** Li Yuan, Mengmeng Zhou, Harpreet S. Wasan, Kai Zhang, Zhaoyi Li, Kaibo Guo, Fengfei Shen, Minhe Shen, Shanming Ruan

**Affiliations:** ^1^The First Clinical Medical College of Zhejiang Chinese Medical University, No. 548, Binwen Road, Binjiang District, Hangzhou 310053, Zhejiang, China; ^2^Department of Traditional Chinese Medicine, The First People's Hospital of Quzhou, No. 2, Zhongloudi Road, Kecheng District, Quzhou 324000, Zhejiang, China; ^3^Department of Cancer Medicine, Hammersmith Hospital, Imperial College Healthcare NHS Trust, London W12 0HS, UK; ^4^Department of Medical Oncology, The First Affiliated Hospital of Zhejiang Chinese Medical University, No. 54, Youdian Road, Shangcheng District, Hangzhou 310006, Zhejiang, China

## Abstract

Colorectal cancer (CRC) is one of the most common malignant tumors affecting the digestive tract. Moreover, the invasion and metastasis of CRC are the main reason therapy is usually inefficient. Decreased intercellular adhesion and enhanced cell motility induced by epithelial-mesenchymal transition (EMT) provide the basic conditions for the invasion and metastasis of the epithelial tumor cells of CRC. The Jiedu Sangen Decoction (JSD) is a prescription that has been used for more than 50 years in the treatment of CRC in the Zhejiang Hospital of Traditional Chinese Medicine. The aim of this study was to investigate the mechanism of JSD-triggered inhibition of invasion and metastasis in colon cancer. In vitro, the EMT model of the SW480 cells was induced by using epithelial growth factor (50 ng/mL). In vivo, the murine model of liver metastasis was constructed by inoculating mice with the SW480 cells. The effects of JSD on cell migration, invasion, and proliferation were determined using the transwell assay and CCK-8 assay. Moreover, the proteins related to the EMT process and the Hippo signaling pathway in the cancerous tissues and cell lines were determined by western blotting and immunostaining. JSD could significantly inhibit the proliferation, migration, and invasion of CRC cells and reverse their EMT status (all, P < 0.05). Moreover, after intervention with JSD, the levels of E-Cadherin (E-cad) increased, whereas the expression levels of N-Cadherin (N-cad), Yes-associated protein (YAP), and the transcriptional coactivator with the PDZ-binding motif (TAZ) decreased in both the SW480 cells and the tumor tissues. In summary, JSD reversed EMT and inhibited the invasion and metastasis of CRC cells through the Hippo signaling pathway.

## 1. Introduction

Colorectal cancer (CRC) is the third most common malignancy, as well as the fourth leading cause of cancer mortality worldwide, with a huge economic and social burden [1, 2]. It is estimated that by 2030, the global burden of CRC will increase by 60% [3]. Invasive metastasis has been the most important cause of treatment failure of CRC. Additionally, the five-year survival rate of patients with distant metastasis in stage III and IV is only about 10% [4]. At present, the therapeutic strategies of metastatic CRC are mainly surgery, radiotherapy, chemotherapy, biological-targeted drug therapy, and treatment using traditional Chinese medicine (TCM). Among these, treatment with TCM has unique advantages in reducing the side effects associated with tumor treatment, improving the quality of life, and prolonging the survival time. This option, therefore, has attracted increasing attention.

TCM is a traditional system of medicine in China, with a legacy spanning thousands of years. Moreover, TCM has the characteristics of being multichannel, multitarget, and multilink in the treatment of tumors. Therefore, it is an important part of current antitumor therapeutic strategies [5, 6]. The Jiedu Sangen Decoction (JSD) is a prescription that has been used for more than 50 years in the treatment of CRC in the Zhejiang Hospital of Traditional Chinese Medicine. It consists of* Actinidia arguta* Siebold & Zucc.,* Adina fauriei *H. Lév., and* Polygonum cuspidatum* Siebold & Zucc. [7, 8]. Furthermore, studies have also confirmed that JSD can inhibit the invasion and metastasis of CRC cells. However, the specific mechanism involved has not yet been clarified.

Epithelial-mesenchymal transition (EMT) is a biological process that is closely related to tumor invasion and metastasis [9]. During EMT, the expression of E-Cadherin (E-cad), which represents the epithelial phenotype, is downregulated. In contrast, the expression of N-Cadherin (N-cad), which represents the stromal phenotype, is upregulated. These two processes lead to the loss of adhesion between tumor cells and thus enhance the metastasis and invasion of cells associated with the development of malignant tumors [10–12].

The Hippo pathway plays an important role in regulating the proliferation and maintenance of stem cells [13, 14]. The Yes-associated protein (YAP) and its paralog, the transcriptional coactivator with PDZ-binding motif (TAZ), are the main downstream effectors of this pathway [15]. YAP/TAZ has proven to be highly expressed in cancers of the colon, liver, ovaries, and lungs [16–18]. Moreover, the inactivation of the Hippo pathway leads to the overexpression of YAP/TAZ. Recent studies have revealed that the Hippo pathway may be involved in the EMT process [19, 20]. In our previous preliminary study, it was observed that JSD could inhibit the invasion and metastasis of the CRC cells [21]. Hence, the aim of the present study was to further investigate whether the Hippo pathway could be the key mechanism involved in the process by which JSD regulates EMT in CRC.

## 2. Materials and Methods

### 2.1. Cell Culture

The SW480 cell line was purchased from the cell bank of the Chinese Academy of Sciences, Shanghai. It was then cultured in the RPMI 1640 medium (Kino Biological and Pharmaceutical Technology Co., Ltd, Hangzhou, China), which was supplemented with 10% fetal bovine serum (FBS) (Gibco, Grand Island, NY, USA) and 1% penicillin/streptomycin (Kino Biological and Pharmaceutical Technology Co., Ltd, Hangzhou, China). The cell lines were cultivated at 37°C under a humidified atmosphere with 5% CO_2_.

### 2.2. JSD Preparation

The medicinal materials of the JSD, including* Actinidia arguta* Siebold & Zucc. (Cat No. 20160721),* Adina fauriei* H. Lév. (Cat No. 20160810), and* Polygonum cuspidatum* Siebold & Zucc. (Cat No. 20160702), were purchased from the pharmacy of the Zhejiang Provincial Hospital of TCM (Zhejiang, China). The place of origin of these three materials is Zhejiang.* Actinidia arguta* Siebold & Zucc. (100 g),* Adina fauriei* H. Lév. (100 g), and* Polygonum cuspidatum* Siebold & Zucc. (100 g) were mixed and immersed in 1000 mL of distilled water for 30 min. The filtrates were concentrated to 150 mL to obtain the JSD, such that its crude drug content was 2 g/mL of mother liquor. The JSD was prepared at the China Pharmaceutical University (Jiangsu, China).

### 2.3. EMT Model Induced by EGF

Once they entered the logarithmic growth phase, the cells were harvested and seeded into culture vessels at a density of 1 × 10^6^ cells/well. They were then cultured at 37°C with 5% CO_2_. After cell attachment, the epidermal growth factor (EGF; 50 ng/mL; Minneapolis, MN, USA) was added to each well prior to incubation for 48 h [22, 23].

### 2.4. Cell Viability Assay

The cell proliferation was evaluated by the CCK-8 assay (Nanjing KeyGen Biotech Co., Ltd.). The cells were seeded in 96-well plate cells (1 × 10^4^ cells/well). When the cells grew to a confluence of 60%, the culture medium was replaced with JSD at different concentrations. After 48 h of incubation, the CCK-8 reagent was added to the culture medium and incubated with the cells for an additional 2 h. The absorbance was measured using a microplate reader (Bio-Rad Laboratories, Inc.) at 450 nm. Meanwhile, the proliferation inhibition rates (%) = (the average OD value in all the duplicates in the control group - the average OD value in the medicine groups)/the average OD value in the blank control group × 100%.

### 2.5. Scratch Test

The experimental groups consisted of the control, EMT, and JSD groups. The EMT and JSD groups were treated with 50 ng/mL EGF for 48 h. The JSD group was then treated with 6 mg/mL JSD for 48 h. Firstly, 2×10^5^ cells were added to the 6-well plates. After treatment with EGF, the cells were subjected to an intervention with JSD for 48 h. Subsequently, scratches were made onto the plates with 200 *μ*L pipette tips. Finally, 3–5 fields of vision were randomly selected, and photos at 0 and 24 h were taken under the optical microscope (100×; IX71, Olympus, Japan).

### 2.6. Transwell Migration and Invasion Assays

In the migration assay, the cell density was adjusted to 2 × 10^6^/mL (the drug interventions were the same as those in the scratch test). Next, the cells in the RPMI 1640 medium (200 *μ*L) were placed into the upper transwell chamber (Corning, New York, NY, USA), whereas the lower chamber was filled with the serum-containing RPMI 1640 medium (600 *μ*L). Following incubation for 48 h, the invaded cells were fixed with 4% paraformaldehyde (Boster Company, Wuhan, China) for 20 min. After staining with 0.1% crystal violet (Shanghai Sangon, China), the invaded cells were counted using a light microscope (200×; IX71, Olympus, Japan). For the invasion assay, 40 *μ*L of diluted Matrigel glue was spread in the upper chamber. Subsequently, the same procedure as above was followed for the migration assay.

### 2.7. Western Blot Analysis

The total amount of protein extracted from the cells and tissues was determined using the RIPA lysis buffer (the drug interventions were the same as those in the scratch test). The concentration of protein was measured using the BCA Protein Assay Kit (Beyotime, Shanghai, China). Next, the proteins (20 *μ*g) were separated using SDS-PAGE (10% gel) and transferred onto a polyvinylidene fluoride (PVDF) membrane (Millipore, MA, USA). After blocking with TPBS supplemented with 5% skimmed milk powder for 1 h, the target proteins in the membranes were probed with the primary antibodies at 4°C, overnight. Subsequently, the membranes were exposed to the corresponding HRP-conjugated secondary antibody for 1 h. The protein blots were visualized using an enhanced ECL (Thermo-Pierce, Rockford, IL, USA) detection method. The antibodies used were as follows: Abcam (Cambridge, MA, USA): N-cadherin antibodies (Art. No. 76011, 1:500); E-cadherin rabbit mAb (Art. No. 76319, 1:1000); Proteintech (Chicago, IL, USA): YAP polyclonal antibodies (Art. No. 13584-1-AP, 1:1000); TAZ polyclonal antibodies (Art. No. 23306-1-AP, 1:1000); *β*-actin antibodies (Art. No. 20536-1-AP, 1:1000); and peroxidase-conjugated goat anti-mouse IgG (H+L) (Art. No. DW0990; 1:1,000), peroxidase-conjugated goat anti-rabbit IgG (H+L) (Art. No. DW-GAR007; 1:1,000) were purchased from Hangzhou Dawen Biology Co., Ltd.

### 2.8. Liver Metastasis of Colon Cancer in Nude Mice

All the animal experiments were approved by the Institutional Animal Care and Use Committee of the Zhejiang Chinese Medical University. The procedures were conducted according to the protocol for experimentation with animals (NIH Publication No. 85–23, revised 1996). A total of 20 male BALB/c (nu/nu) mice (15 ± 1 g, 4 weeks old) were obtained from the Vital River Laboratories of Beijing (Beijing, China). Briefly, the mice were housed in the Specific Pathogen Free (SPF) barrier center at the animal experimental center of the Zhejiang Chinese Medical University, under standard conditions of temperature (25 ± 2°C) and humidity (50 ± 5%) in a 12-h light/12-h dark cycle and fed normal food and drink.

Subsequently, the SW480 cells were collected during the logarithmic growth phase, the concentration of which was adjusted to 3 × 10^7^/mL. In order to establish the liver metastasis model of CRC, a cell suspension (0.1 mL) was injected into the spleen membrane of each nude mouse as previously described [24, 25]. After 2 weeks, the conditions of the animal model were detected by a live fluorescence imaging technique. Based on live imaging results (grouped according to signal intensity), the eligible mice were divided into the blank control group and the JSD group (10 mice per group). Briefly, the mice in the JSD group were fed with JSD (1.2 kg/L, 0.4 mL/20 g) once a day for 2 weeks. In contrast, the mice in the blank control group were given normal saline. Liver metastasis was detected using a live fluorescence imaging technique. The nude mice were then sacrificed by cervical dislocation and the tumor tissues were taken for hematoxylin-eosin and western blot experiments.

### 2.9. Bioluminescence Imaging

In vivo, the bioluminescence imaging was determined using the cooled CCD camera system (PerkinElmer, CA, USA). Before imaging, the mice were intraperitoneally injected with D-luciferin (15 mg/mL, Yeasen Corp., Shanghai, China; 150 mg/kg). They were then placed in a light-tight chamber with 2% isoflurane anesthesia in the air. The mice were imaged on day 14 (grouped according to signal intensity) and day 28 (with efficacy evaluated according to signal intensity), respectively. For the luminescent image acquisition, an integration time of 1 to 60 s and a binning factor of 4 was used. The signal intensity was measured according to the flux of all the detected photon counts from the region of interest prescribed over the tumor area using the Living Image software package (Xenogen Corp., Alameda, CA, USA).

### 2.10. Hematoxylin-Eosin (HE) Staining and Immunohistochemistry

Paraformaldehyde-fixing, ethanol dehydration, transparency with xylene, and paraffin-embedding procedures were carried out for all tissues. HE (Art. No. ZLI-9609 ZSGB-BIO Corp., Shanghai, China) staining was used to stain the tissue slices. The histological changes in the tumor tissues were observed with a microscope at 200× magnification.

For immunohistochemistry staining, 4-*μ*m tissues slides were treated with 1 mM EDTA buffer (pH = 9.0) for antigen retrieval. The samples were incubated with the primary antibodies. They were then incubated with biotin-labeled goat-rabbit IgG and horseradish peroxidase-conjugated streptavidin for 1 h. The following antibodies were used: N-Cadherin antibodies (Art. No. 76011, 1:500); E-Cadherin rabbit mAb (Art. No. 76319, 1:300); YAP polyclonal antibodies (Art. No. 13584-1-AP, 1:300); TAZ polyclonal antibodies (Art. No. 23306-1-AP, 1:300), and a hypersensitive enzyme-labeled goat anti-mouse/rabbit IgG polymer (Art. No. PV-8000,1:1). The immunoreaction was visualized by diaminobenzidine (DAB; Art. No. ZLI-9065, ZSGB-BIO Corp., Shanghai, China). It was then photographed with an inverted microscope at 200× magnification. Image-Pro Plus Version 6 software was used for the analysis of the total integral optical density (IOD) and the mean density (MD) of the positive area.

### 2.11. Statistical Analysis

All the statistical analyses were performed with SPSS 22.0 software (IBM, New York, NY, USA). All data are represented as the mean ± standard deviation ($\overset{-}{X}\pm s$). One-way ANOVA followed by Tukey's multiple comparison test was performed when the data followed a normal distribution. Additionally, Kruskal Wallis H test was used when the data did not follow a normal distribution.* P* < 0.05 was considered as statistically significant. All the experiments were repeated in triplicate.

## 3. Results

### 3.1. JSD-Induced Inhibition Effects on the Proliferation of the CRC Cells In Vitro

**Figure 1:**
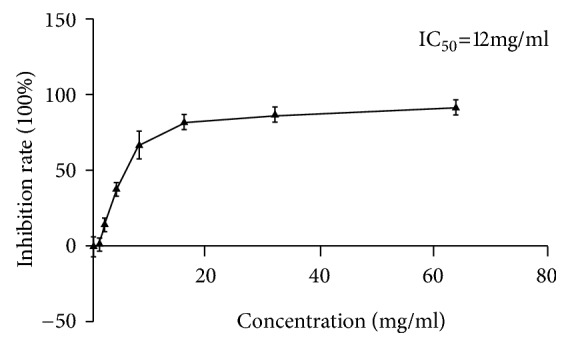
JSD inhibited the proliferation of colon cancer cells in vitro. The cells were treated with JSD at concentrations of 0, 1, 2, 4, 8, 16, 32, and 64 mg/mL, respectively. The inhibitory effects were enhanced with an increase in the dose. Meanwhile, according to our calculations, the half inhibitory concentration (IC50) of JSD was 12 mg/mL in the SW480 cells; n = 9.

To observe the inhibitory effect of JSD on the proliferation of the CRC cells, different concentrations (0, 1, 2, 4, 8, 16, 32, and 64 mg/mL) of JSD were used to interfere with the SW480 cells for 48 h. As shown in Figure 1 and Table 1, the results of the CCK-8 assay revealed that the inhibitory effects of JSD on the SW480 cell proliferation were positively correlated with its concentration. Meanwhile, according to our calculations, the half inhibitory concentration (IC_50_) of JSD was 12 mg/mL in the SW480 cells. Therefore, 6 mg/mL was used for the subsequent experiments, to observe the effect of JSD on the invasion and metastasis of the SW480 cells.

### 3.2. JSD-Induced Inhibitory Effects on the Migration and Invasion Ability of the CRC Cells In Vitro

**Figure 2:**
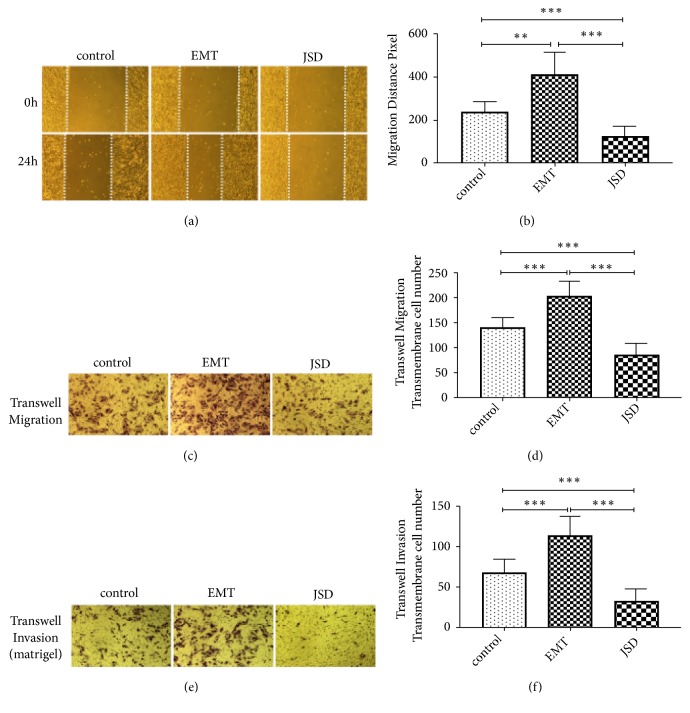
The JSD-induced inhibitory effects on the migration and invasion ability of the colon cancer cells in vitro. The experimental group consisted of control, EMT, and JSD. The EMT and JSD groups were treated with 50 ng/mL EGF for 48 h. Next, the JSD group was treated with 6 mg/mL JSD for 48 h. ((a)–(b)) Compared with the control group, the migration distance of the EMT group was significantly increased (*P* < 0.01), whereas the migration distance of the JSD group was remarkably decreased (*P* < 0.001). In addition, the migration distance of the JSD group was significantly shorter than that in the EMT group (*P *< 0.001). ((c)–(f)) The number of transmembrane cells in the EMT group was observed to be upregulated in comparison with that in the control group (both,* P* < 0.001), whereas the number of transmembrane cells in the JSD group was significantly downregulated in comparison with the control group (both,* P* < 0.001). At the same time, compared with the EMT group, the number of transmembrane cells in the JSD group was significantly reduced (both,* P* < 0.001). *∗∗ P* < 0.01, *∗∗∗ P* < 0.001; n = 9.

As illustrated in Figures 2(a) and 2(b), the results suggested that, in comparison with the control group, the migration distance of the EMT group was significantly increased (*P < *0.01). In contrast, the migration distance of the JSD group was remarkably decreased (*P < *0.001). In addition, the migration distance of the JSD group was significantly shorter than that in the EMT group (*P < *0.001).

Similar to the scratch test, the results of the transwell migration and invasion assays revealed that the number of transmembrane cells in the EMT group was observably increased in comparison with that in the control group (both,* P < *0.001; Figures 2(c)–2(f)). In contrast, the number of transmembrane cells in the JSD group was significantly decreased in comparison with the control group (both,* P < *0.001). At the same time, compared with the EMT group, the number of transmembrane cells in the JSD group was significantly reduced (both,* P < *0.001). All these results suggested that JSD could inhibit the migration and invasion as well as reversing the EMT status of the CRC cells.

### 3.3. The JSD-Induced Reversion of EMT Progression Depends on the Activation of Hippo Signaling

**Figure 3:**
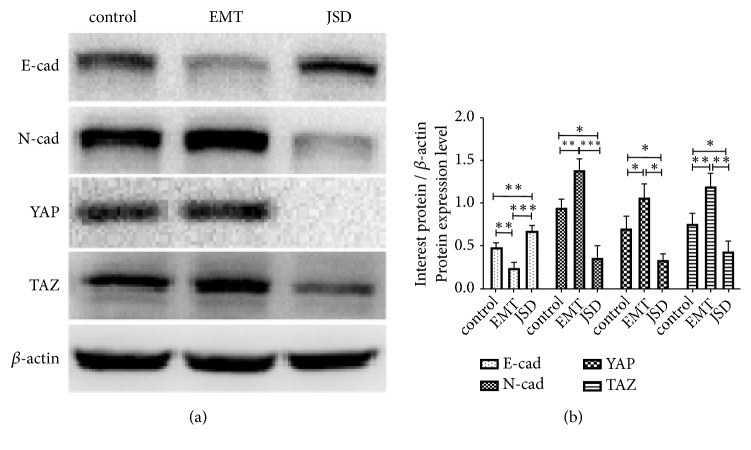
Proteins related to the progression of EMT and the activation of the Hippo signaling in vitro. (a) Pictures of the western blot assays. (b) Relative expression of E-cad, N-cad, YAP, and TAZ. Compared to the control group, the levels of E-cad were decreased, whereas those of N-cad were increased in the EMT group (both,* P* < 0.01). Additionally, YAP and TAZ levels in the EMT group were observably higher than those in the control group (both,* P* < 0.05), respectively. On the contrary, E-cad was significantly upregulated in the JSD group in comparison with that in the control group (*P* < 0.01). However, N-cad, YAP, and TAZ were downregulated (all,* P* < 0.05). *∗ P* < 0.05, *∗∗ P* < 0.01, *∗∗∗ P* < 0.001; n = 3.

To explore the potential mechanism of the intervention of JSD on the progression of EMT, the related proteins and the Hippo pathway were analyzed by western blotting. The results revealed that compared to the control group, the expression of E-cad was decreased, whereas that of N-cad was increased in the EMT group (both,* P* < 0.01; Figure 3). Additionally, the YAP and TAZ levels in the EMT group were observably higher than those in the control group (both,* P* < 0.05), respectively. On the contrary, E-cad was significantly upregulated in the JSD group in comparison with that in the control group (*P* < 0.01). However, N-cad, YAP, and TAZ were downregulated (all,* P* < 0.05). The results suggest that JSD might reverse the EMT status of the colon cancer cells through the regulation of the Hippo pathway.

### 3.4. JSD-Induced Inhibition of CRC Liver Metastasis In Vivo

**Figure 4:**
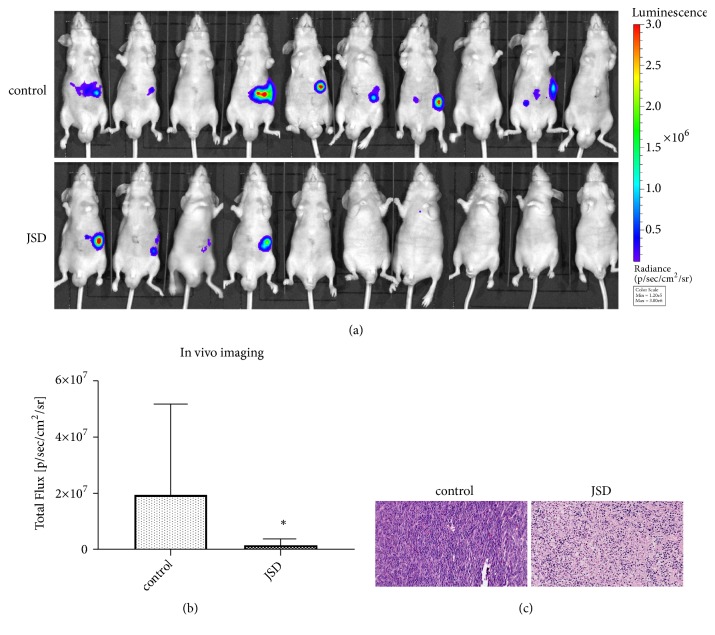
The JSD-induced inhibitory effects on the colon cancer liver metastasis in vivo. (a) Colon cancer liver metastases of mice observed by a live fluorescence imaging technique. (b) The total flux of fluorescence signals in the mice. (c) HE staining in the tumor foci of the mice. *∗ P* < 0.05; n = 10.

The liver metastasis in mice was evaluated with a live fluorescence imaging technique. As shown in Figure 4(a), 7 of 10 nude mice in the control group had liver metastases, whereas only 4 of 10 nude mice in the JSD group did. Meanwhile, in comparison with the control group, the total fluorescence value of the JSD group was significantly reduced (*P = *0.023; Figure 4(b)).

In addition, the results of the HE staining revealed that the shape of the tumor cells in the control group was irregular and the nuclei were hypertrophic, abnormal, and deeply stained (Figure 4(c)). However, the density of the tumor cells in the JSD group was significantly reduced. Additionally, the number of the atypical cells was significantly reduced.

### 3.5. JSD-Induced Reversion of EMT Progression Depends on the Hippo Signaling Activation In Vivo

**Figure 5:**
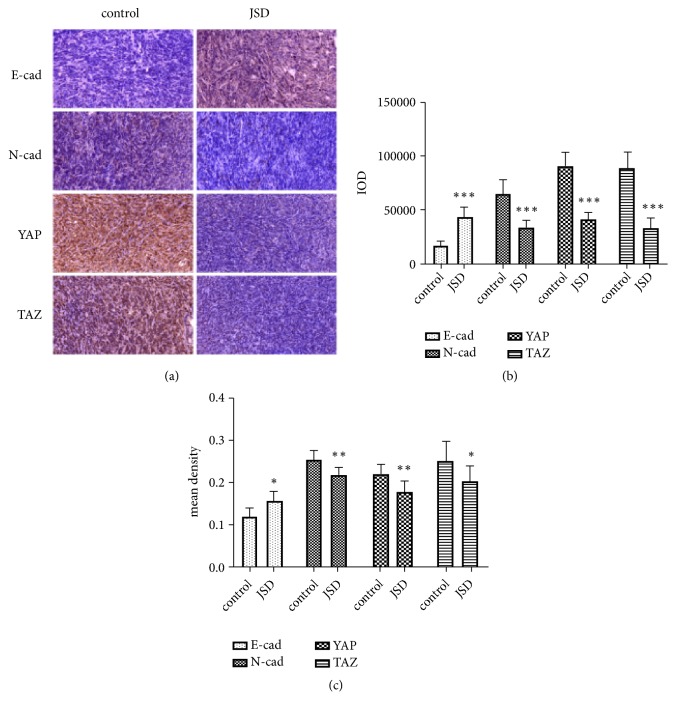
The JSD-induced reversion effects on the progression of EMT depending on the activation of the Hippo signaling in vivo. (a) Immunohistochemical staining assays. (b) Relative expression of the objective proteins (IOD). (c) Relative expression of the objective proteins (mean density). Compared with the control group, E-cad expression was significantly increased in the JSD group with higher IOD and (*P *< 0.001) and mean density (*P* < 0.05), whereas the expressions of N-cad, YAP, and TAZ in JSD group were all observably lower than those in the control group. *∗ P* < 0.05, *∗∗ P* < 0.01, *∗∗∗ P* < 0.001; n = 6.

Immunohistochemical staining was used to further verify the expression profiles of E-cad, N-cad, YAP, and TAZ in the tumor tissues of nude mice. The data revealed that in comparison with the control group, the expression of E-cad was significantly increased in the JSD group (Figure 5(a)) with a higher IOD (*P < *0.001; Figure 5(b)) and mean density (*P < *0.05; Figure 5(c)). Additionally, the expressions of N-cad, YAP, and TAZ in the JSD group were all observably lower than those in the control group (all,* P < *0.05).

**Figure 6:**
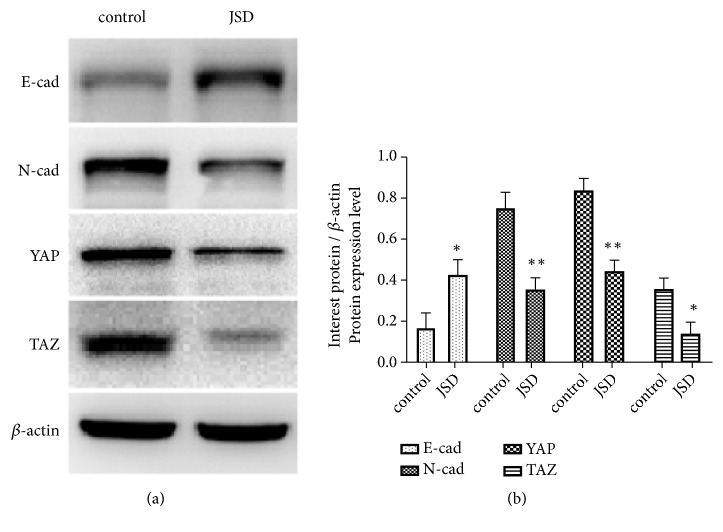
The JSD-induced reversion effects on EMT progress depend on the Hippo signaling activation in vivo. (a) Western blot assays in the tumor tissues. (b) Relative expression of the objective proteins. Compared to the control group, the expression of E-cad was significantly increased in the JSD group (*P* < 0.05). Also, N-cad (*P* < 0.01), YAP (*P* < 0.01), and TAZ (*P* < 0.05) were significantly reduced in the JSD group. *∗ P* < 0.05, *∗∗ P* < 0.01; n = 3.

Similar to the immunohistochemical assay, the results of western blotting revealed that compared to the control group, the expression of E-cad was significantly increased in the JSD group (*P < *0.05; Figure 6). Meanwhile, in the same group, the N-cad (*P* < 0.01), YAP (*P* < 0.01), and TAZ (*P* < 0.05) expression were significantly reduced. These results further demonstrate that JSD could reverse the EMT status of CRC cells through the regulation of the Hippo pathway.

## 4. Discussion

JSD is well known to be involved in the invasion and metastasis of CRC cells. However, the specific mechanism of action remains unclear. In the present study, based on the EGF-induced EMT model, it was revealed that JSD could significantly inhibit the proliferation, migration, and invasion of CRC cells. Additionally, it could reverse the EMT status of the CRC cells by regulating the Hippo pathway. Moreover, the in vivo experiments, i.e., the immunohistochemical and western blotting assays, revealed that JSD induced an inhibitory effect on the colon cancer liver metastasis through the regulation of the protein levels related to the EMT process and the Hippo pathway.

E-cad is a key component of the adhesion junction, which is essential for cell adhesion and the maintenance of the epithelial phenotype. Hence, the loss of E-cad has been regarded as the key to EMT initiation [26, 27]. On the contrary, N-cad is an important marker of the mesenchymal-like cells and its levels are elevated in the EMT process [28, 29]. In this study, 50 ng/mL of EGF was used to induce the EMT model of the SW480 cells. The results revealed that the expression level of the E-cad protein was downregulated, whereas that of the N-cad protein was upregulated in the SW480 cells of the EMT group. Furthermore, the expression of the E-cad protein was upregulated, whereas that of the N-cad protein was downregulated after the intervention with JSD. Similarly, in vivo, it was demonstrated that JSD could inhibit the formation of liver metastases from colon cancer in nude mice. Additionally, JSD could increase the levels of E-cad and decrease the levels of N-cad in the tumor tissues. Thus, these results indicate that JSD could reverse the EMT status of CRC.

The decreased intercellular adhesion and the enhanced cell motility induced by EMT provide the basic conditions for the invasion and metastasis of tumor cells [30]. Previous studies have reported that the expression of E-cad is downregulated in the CRC tissues [31], which is negatively correlated with tumor stage, malignancy, and prognosis [32]. Other studies have demonstrated that the upregulation of E-cad and the downregulation of N-cad can significantly inhibit the invasion and metastasis of the CRC cells [33]. Therefore, EMT may be the key to the invasion and metastasis of CRC. In accordance with previous studies, in the present study, it was revealed that JSD could upregulate E-cad and downregulate N-cad. Thus, it could reverse the EMT state of the CRC cells. Moreover, the scratch tests and transwell assays demonstrated that JSD could inhibit the invasion and metastasis of the colon cancer cells in vitro. Therefore, JSD may inhibit the invasion and metastasis of CRC by reversing the EMT status of the CRC cells.

The Hippo pathway is the main signaling pathway that regulates not only cell size and organ volume but also stem cell self-renewal and tissue regeneration. These effects are modulated especially in close relation with the occurrence and development of cancer. YAP/TAZ is the main downstream effector molecule [34], and many studies have shown that YAP/TAZ is highly expressed in CRC tissues and negatively correlated with its prognosis [35–37]. Meanwhile, an increasing number of studies have shown that the Hippo pathway is closely related to the progression of EMT as well as tumor invasion and metastasis. For example, Yu et al. reported that the targeted inhibition of the expression of YAP can inhibit the metastasis of hepatocellular carcinoma and EMT [38]. Jiang et al. demonstrated that the knockout of the YAP gene could inhibit the occurrence of EMT and the invasion and metastasis of pancreatic cancer cells [39]. Furthermore, Shen et al. revealed that the knockdown of TAZ can inhibit osteosarcoma cell proliferation and EMT [40]. In the present study, our results confirmed that the expressions of YAP and TAZ were both upregulated in the EMT status of SW480 cells. After the intervention with JSD, the expressions of YAP and TAZ were markedly inhibited in the CRC cells and tissues. Therefore, JSD may reverse EMT and inhibit the invasion and metastasis of CRC cells by inhibiting the expression of YAP and TAZ.

## 5. Conclusions

In summary, this study revealed that JSD could downregulate YAP and TAZ of the Hippo signaling pathway. Moreover, JSD could inhibit the invasion and metastasis of CRC and reverse the progression of EMT by increasing E-cad and decreasing N-cad levels through the Hippo signaling pathway. In this study, we have revealed the mechanism of JSD on CRC, which will provide a new basis for the treatment of CRC.

## Figures and Tables

**Table 1:** Proliferation inhibition rates (IR) of the SW480 cell line treated with various concentrations of medicine for 48 h (X ± S, n = 9).

Concentration (mg/mL)	0	1	2	4	8	16	32	64
IR (%) in SW480	0.06 ± 6.48	1.45 ± 4.57	14.97 ± 4.14	38.36 ± 4.87	67.42 ± 8.86	82.60 ± 4.91	87.40 ± 4.91	92.55 ± 5.02

## Data Availability

The data used to support the findings of this study are available from the corresponding author upon request.
